# Small Scattered Fragments Do Not a Dwarf Make: Biological and Archaeological Data Indicate that Prehistoric Inhabitants of Palau Were Normal Sized

**DOI:** 10.1371/journal.pone.0003015

**Published:** 2008-08-27

**Authors:** Scott M. Fitzpatrick, Greg C. Nelson, Geoffrey Clark

**Affiliations:** 1 Department of Sociology & Anthropology, North Carolina State University, Raleigh, North Carolina, United States of America; 2 Department of Anthropology, University of Oregon, Eugene, Oregon, United States of America; 3 Archaeology and Natural History, Division of Society and Environment, Research School of Pacific and Asian Studies, Australian National University, Acton, Australia; American Museum of Natural History, United States of America

## Abstract

Current archaeological evidence from Palau in western Micronesia indicates that the archipelago was settled around 3000–3300 BP by normal sized populations; contrary to recent claims, they did not succumb to insular dwarfism.

**Background:**

Previous and ongoing archaeological research of both human burial and occupation sites throughout the Palauan archipelago during the last 50 years has produced a robust data set to test hypotheses regarding initial colonization and subsequent adaptations over the past three millennia.

**Principal Findings:**

Close examination of human burials at the early (ca. 3000 BP) and stratified site of Chelechol ra Orrak indicates that these were normal sized individuals. This is contrary to the recent claim of contemporaneous “small-bodied” individuals found at two cave sites by Berger et al. (2008). As we argue, their analyses are flawed on a number of different analytical levels. First, their sample size is too small and fragmentary to adequately address the variation inherent in modern humans within and outside of Palau. Second, the size and stature of all other prehistoric (both older and contemporaneous) skeletal assemblages found in Palau fall within the normal parameters of modern human variation in the region, indicating this was not a case of insular dwarfism or a separate migratory group. Third, measurements taken on several skeletal elements by Berger et al. may appear to be from smaller-bodied individuals, but the sizes of these people compares well with samples from Chelechol ra Orrak. Last, archaeological, linguistic, and historical evidence demonstrates a great deal of cultural continuity in Palau through time as expected if the same population was inhabiting the archipelago.

**Conclusions:**

Prehistoric Palauan populations were normal sized and exhibit traits that fall within the normal variation for *Homo sapiens*—they do not support the claims by Berger et al. (2008) that there were smaller-bodied populations living in Palau or that insular dwarfism took place such as may be the case for *Homo floresiensis*.

## Introduction

Current archaeological data from Palau (western Micronesia) indicate that prehistoric peoples probably settled the archipelago around ca. 3000–3300 cal BP from somewhere in Island Southeast Asia by groups who practiced agriculture and exploited a wide range of marine resources. Close examination of early human remains from burial sites, most notably Chelechol ra Orrak (B:IR-1:23), but including several others, indicate that these peoples (ca. 2000–3000 BP) were normal sized individuals with biological traits known to occur within this and other populations. This is contrary to the report of “small-bodied” individuals found recently at two cave sites in Palau by Berger et al. [1] suggesting that this or a separate migratory group succumbed to insular dwarfing, with samples showing “small body size, reduction of the absolute size of the face, distinct supraorbital tori (in some individuals), a weakly developed mental eminence, relatively large dental dimensions, and dental dysplasias and agenesis.” As such, they argue that this provides “important insights into the relationship between small body size and the expression of morphological features generally considered to be taxonomically diagnostic of our genus” [1, p. 1].

Our long-term and extensive research on human skeletal series and archaeological assemblages from numerous sites in Palau, in conjunction with previous studies, raises serious doubts, however, concerning the validity of Berger et al.'s [1] claims and the methods they used. Although we have not seen the material that Berger et al. [1] base their results on, we can speak to the diversity and normalcy of human skeletal series from throughout the archipelago that have been excavated from several burial caves over the last decade (see [2]–[4] as well as an abundance of archaeological, linguistic, and historical data indicating a general continuity of cultural traits over a period of three millennia.

The primary conclusion of Berger et al. [1], that rapid reduction in body size in representative populations of the genus *Homo* precipitates “morphological features considered primitive for the genus *Homo* (e.g., small brain size, enlarged supraorbital tori, and absence of chins) or unique to *H. floresiensis* within the genus *Homo* (e.g., relative megadontia)” (p. 9), is not addressed here since, in fact, these characters may indeed occur in populations of *Homo* that have undergone insular dwarfing. Currently however, no prehistoric or living populations of *Homo* have been conclusively shown to have undergone such dwarfing so this hypothesis is unsupported and not testable at this time. Additionally, we do not address the validity of *H. floresiensis* as a taxon. Our principle concern is with the conclusion that a population of extremely small-bodied members of *Homo sapiens* inhabited Palau at any point in time. Physical anthropological evidence collected and reported by two of the authors (SMF and GCN) [2], [3], [5]–[8] and others [4], [9]–[12] indicate that the earliest inhabitants of the archipelago and their descendents possessed skeletal and dental dimensions consistent with normal body size. Archaeological data also do not suggest a separate isolated group evolving differently (biologically or culturally), although there are subtle differences and changes that occur through time (e.g., [4], [9], [13]–[19]).

We contend that Berger et al. [1] misinterpret data derived from very fragmentary remains and reach false conclusions because they lack an understanding or appreciation of the morphological pattern in prehistoric Pacific Island populations. Compounding their errors, they have reached these conclusions without benefit of comparing their data to that readily available on skeletal and dental dimensions derived from early inhabitants of Micronesia and surrounding areas [3], [10], [11], [20]–[28]. Had they done so, they would have seen that features they interpret as indicating reduced body size in early Palauans are actually well within the range of variation for early Oceanic populations of *Homo sapiens*. 

Methodological problems such as those mentioned above stem, we feel, from a fundamental error; that of an incorrect original assumption or null hypothesis. Researchers familiar with Oceanic prehistory should work from a null hypothesis that human skeletal material found in Palau represent modern humans of normal stature and body mass. If, after a reasonable sample had been collected, and thorough comparisons to temporally and spatially contemporary skeletal series completed, the discovered sample appeared to represent a separate population, then other hypotheses could be tested. Unfortunately, it appears that Berger et al. [1] did not do this and instead operated from a null hypothesis, based off their initial impression from a few fragments that exhibited small or primitive dimensions (one of which—apparent brow ridges—turned out to be carbonate precipitate that eventually flaked off) , that their sample represented a population of small-bodied humans.

Here we present preliminary data on early remains from the well-stratified and rigorously dated site of Chelechol ra Orrak that spans the last three millennia, compare them with the data presented by Berger et al. [1], couple these data with cranial measurements of our samples and other populations in the Pacific, and review current knowledge of Palauan prehistory to address Berger et al.'s claims [1].

We first provide a geographical background to emphasize the spatial arrangement of islands and resource availability that has relevance for discussions of insular dwarfism. We then report on newly collected metric and non-metric data from excavations of early burials at Chelechol ra Orrak. These findings are then contextualized with what we know archaeologically and biologically in Palau. We note that the skeletal features seen in these early populations do not appear to indicate that there were small-bodied individuals living in complete or even relative genetic isolation. Overall, we conclude that the results of Berger et al. [1], as they stand, are critically flawed and cannot be accepted without further verification by other researchers (not necessarily us) more familiar with both the morphology of modern humans and the range of variation exhibited by prehistoric Palauans and other contemporary skeletal series of the region.

### Geographical Background

**Figure 1:**
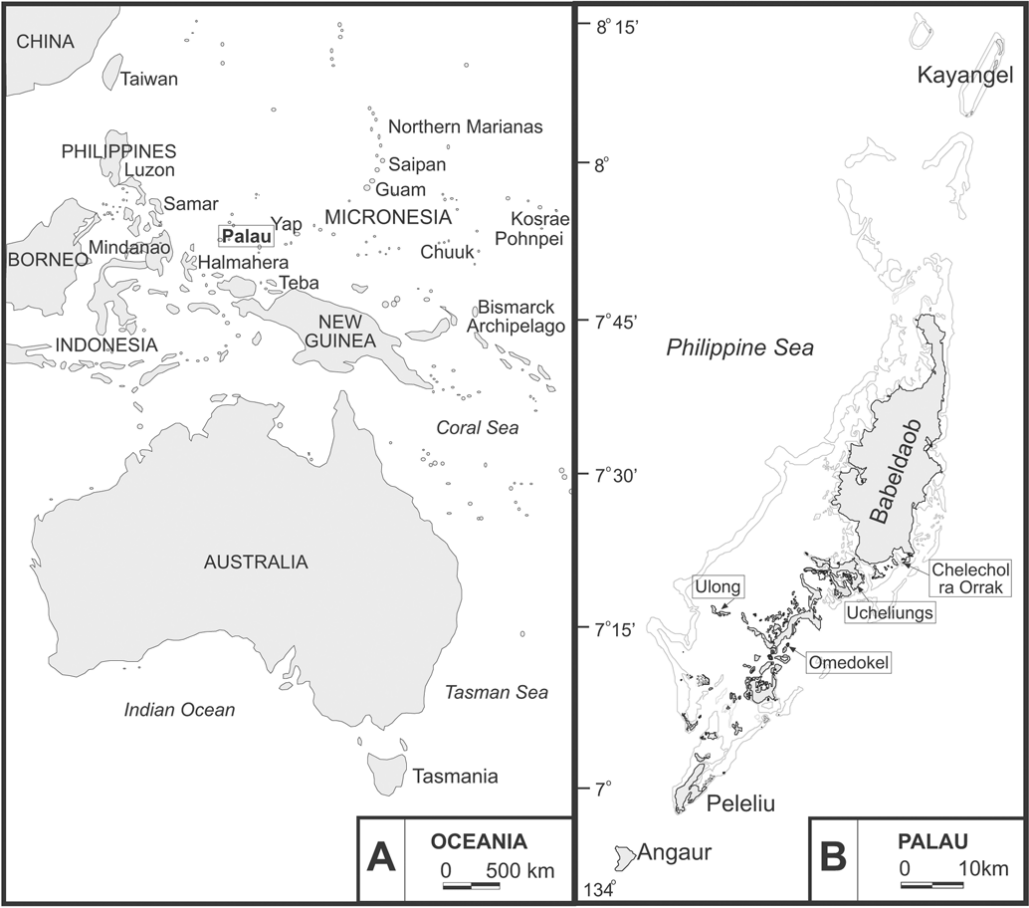
Map of the western Pacific (A) with inset of Palauan archipelago (B) (site names referred to in text are boxed).

Palau is located roughly 600 km equidistant from the Philippines to the west and New Guinea to the south (Figure 1). The main archipelago is situated at 7° 30′ north of the equator and is approximately 160 km long, 25 km across at its widest point, and oriented in a northeast-southwest direction. There are several hundred islands in Palau that include volcanic, coral reef and atoll, and limestone [29] that form a land area of approximately 400 km^2^. The volcanic islands comprise about 80% of Palau and include Babeldaob, Meiuns, Malakal, and the western portion of Koror (Babeldaob is the largest, more than 330 km^2^ in area). The “Rock Islands,” as they are known locally, are the most abundant island type and extend 30 kilometers in length primarily between Peleliu and Koror. Kayangel and Ngaruangel are small atolls north of Babeldaob [30], the only ones which are not entirely intervisible from one island to another. The Southwest Islands (Merir, Sonsorol, Tobi, and Helen Reef), a political addition to the Republic but linguistically and culturally distinct, are comprised of low platform islands and atolls, while Peleliu and Angaur are considered low platforms.

The Palauan coral ridge consists of a well-developed barrier and fringing reef that surrounds most of the inner Rock Islands except for the two smaller atolls and the island of Angaur in the south. The barrier reef forms a breakwater to the outside ocean currents and encloses a lagoon that ranges in depth from a few centimeters of surface water to 40 m or more [31]. Fringing reefs border many of the individual islands, and reef ridges and mounds are abundant in the lagoon and passages between the islands and the barrier reef. These reefs within the lagoon range from a few meters to more than several kilometers wide [30].

The Palau Islands support an extensive array of marine and aquatic habitats that include the atolls, barrier reefs, fringing reefs, patch reefs, reef walls, lagoons, pinnacles, passes and channels, mangrove forests, sea grass flats, mud flats, sand and rubble flats, emergent limestone islets, estuaries, freshwater streams, blue holes and submerged tunnels, and marine and freshwater lakes [32]–[35]. The reef and coastal systems of the main archipelago and the Southwest Islands have been described in detail by Maragos and Meier [36] and Maragos et al. [32].

The islands support the most diverse stony coral, marine fish, and freshwater fish faunas in Micronesia [35]–[38]. Marine plants and most invertebrate groups enjoy similar levels of diversity [36]. The islands of Palau contain approximately 1500 species of fish, four marine turtles, dugong, saltwater crocodile, 120 genera of algae and seagrasses, 230 species of crab, 300 mollusk species, 120 echinoderms, 400 hard corals, and 100 ascidian (sea squirt) species [39].

### Archaeological Background

Archaeological evidence collected over the past 50 years (e.g., [6], [8], [9], [13], [15]–[17], [19], [40]–[49] from a number of different site types, including human burials [2], [3], [5], [7], [50], suggests that Palau was colonized sometime between 3000–3300 BP [5], [7], [15], [47], [51]. Based on mtDNA lineage analyses [52], multivariate analysis of craniometric data [10], artifactual evidence [15], and computer simulations of voyaging [53], [54] it appears that the earliest colonists originated from somewhere in Island Southeast Asia, possibly the Philippines, more than 400 nautical miles (600 km) from Palau, although migration from elsewhere such as Melanesia cannot be entirely discounted.

A number of archaeological sites and human remains in Palau have been radiocarbon dated back to ca. 2700–3300 BP. These include a habitation site on the small island of Ulong along the western edge of the barrier reef [15], [47]and several human burial sites in limestone caves and rockshelters, including Chelechol ra Orrak [3], [7], Ngermereues Ridge [4], and the human remains from Ucheliungs and Omedokel reported by Berger et al. [1]. Paleoenvironmental [55]–[57] and paleoshoreline [58] data suggest that colonization may have taken place 1000–1500 years earlier, but to date no firm archaeological evidence supports this longer chronology and it is of limited relevance to our criticisms of the claims advanced by Berger et al. [1].

## Analysis

### Archaeological Investigations at Chelechol ra Orrak (B:IR-1:23)

**Figure 2:**
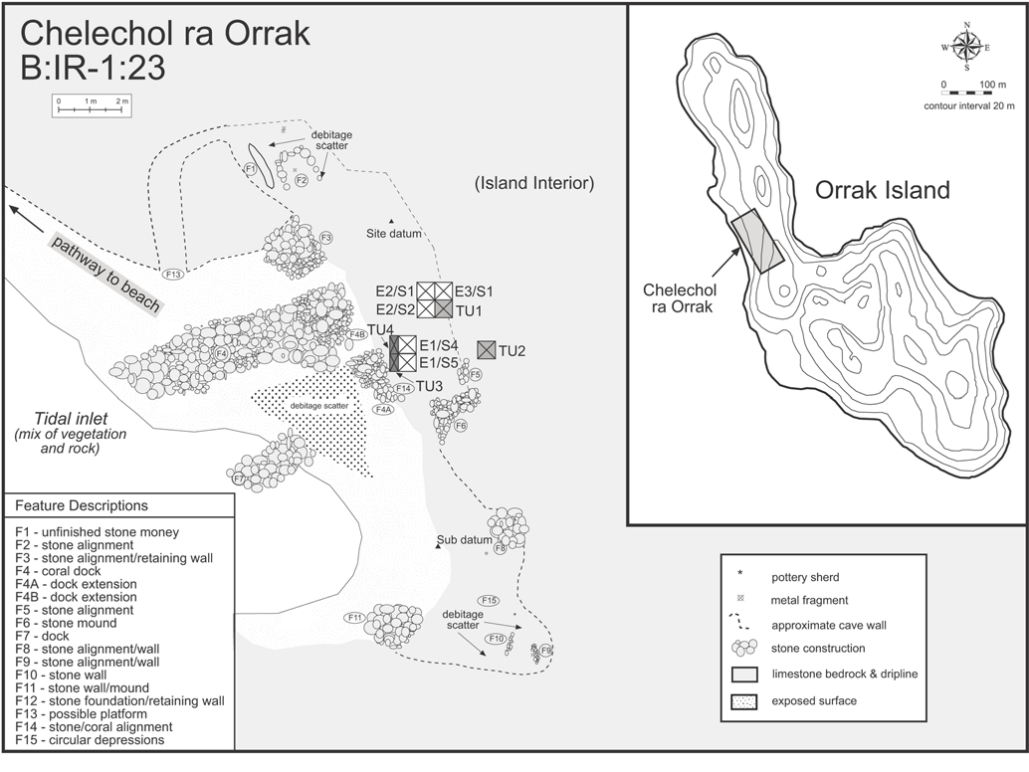
Map of Chelechol ra Orrak (B:IR-1:23).

**Figure 3:**
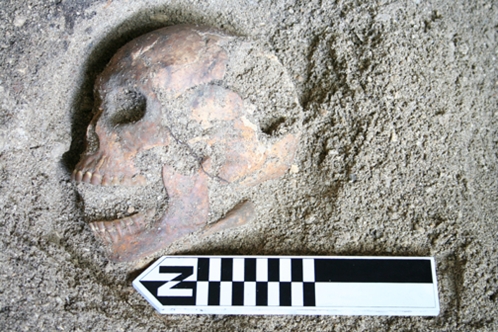
One of several human skulls found in unit E3/S1 at Chelechol ra Orrak in 2007 (depth = approximately 85–90 cmbs); all are normal sized (photo by SM Fitzpatrick).

In contrast to other limestone cave and rockshelter contexts in Palau, including Ucheliungs and Omedokel caves, the cemetery at Chelechol ra Orrak (‘beach of Orrak’) has the most extensive collection of early human remains yet found in Palau and are some of the oldest in Remote Oceania, dating to ca. 3000 BP [see also 59]. The site was first intensively investigated by Fitzpatrick in 2000 with Palauan archaeologists from the Bureau of Arts and Culture. Initial excavation involved opening up two 1×1 m and two 1×0.5 m units that reached depths of 1 m (Figure 2). The skeletal assemblage, discovered at around 50 cm deep underlying, and pre-dating, occupation levels [7], consisted of approximately 25 individuals comprising prenates, neonates, adolescents, and adults of both sexes [3]. Fitzpatrick and Nelson continued work at the site in 2002 by excavating three additional 1×1 m units (E2/S1, E3/S1, E2/S2) to depths of 40–50 cm. In 2007, Fitzpatrick excavated these three units down to a depth of 1.0 to 1.1 m along with two additional 1×1 m units (E1/S4, E1/S5). Although many of the skeletal remains are fragmentary, several nearly complete, articulated, and well-preserved individuals have been recorded (though not all recovered), most of which were buried in a supine position and have relatively complete crania (Figure 3).

### Estimations of Size and Stature

At Chelechol ra Orrak, we have, to date, recovered one nearly complete skeleton, several crania, and numerous whole bones that combine the small facial and articular dimensions reported by Berger et al. [1] with crania and long bones of people of normal size and height for this region. As can be seen in Table 1, femoral head diameters equal to or smaller than those reported by Berger et al. [1] belong to individuals who would be considered to be of normal height, particularly for peoples practicing a hunter gatherer lifeway with a developing horticultural base. Berger et al. [1] base all their conclusions on fragmentary remains—as such, they must extrapolate adult body size, mass, and inferred height from incomplete specimens that may not reflect the true morphology of the individuals sampled.

**Table 1:** Femoral dimensions (mm) of specimens from Chelechol ra Orrak and Omedokel Cave [1].

Site	Specimen	Head Antero-posterior Diameter	Head Supero-inferior Diameter	Head Maximum Diameter	Maximum Length
Orrak	B:IR-1:23-001-L	36.9	37.1	37.2	412
Orrak	B:IR-1:23-001-R	36.6	36.8	36.9	411
Orrak	B:IR-1:23-002-L	44.4	44.3	44.9	–
Orrak	B:IR-1:23-003-L	–	–	38.5	392
Orrak	B:IR-1:23-003-R	–	–	36.1	–
Omedokel	B:OR-15:18-013	–	35.2	–	–
Omedokel	B:OR-15:18-098	38.8	–	–	–

Note: The supero-inferior head diameter for specimen B:OR-15:18-013 recorded in this table (35.2 mm) is the measure that Berger et al. [1] report in the text (p. 5). However in their Table 1 and supplementary data in Table 4, they report an Antero-posterior diameter of 36.1 mm which is used to calculate the mean. In the text, they report a biomechanical neck length for this specimen of 36.1 mm. It appears they have inadvertently transformed this neck length measure into an antero-posterior head diameter in their Table 1 and supplementary data 4 Table and then used it in their calculations.

When mean values are calculated, those reported by Berger et al. [1] for femoral head diameter (37.5±1.9 mm) are only slightly less than for those from Orrak (mean A-P diameter of 39.3±4.4 mm; S-I diameter of 39.4±4.2 mm). Because of the unreliability of the mean reported by Berger et al. [1] for femoral head diameter (see note below Table 1) and that Nelson and Fitzpatrick [3] report only the maximum head diameter for specimen -003, we compare the mean for maximum femoral head diameter from Orrak to that from Omedokel. When the large individual (-002) is removed, the mean for Orrak falls to 37.16±1.0 mm, meaning that the average *maximum* femoral head diameter for two individuals from Orrak is below that reported by Berger et al. [1] As can be seen by the femoral lengths recorded for these individuals (an average length of 405±11.3 mm which equates to an adult height of between 152 and 157 cm depending on formulae used [see 60], this is well within the expected range for modern human adult females which both of these individuals appear to be. Beyond femora, for all dimensions reported by Berger et al. [1] for other skeletal elements, the material from Orrak falls within the one standard deviation [61]. Some are smaller (tali) and some are larger (tibiae), but all fit a pattern in which apparently small articular dimensions belong to normal sized individuals when the total morphological pattern is considered. It is easy to see how Berger et al. [1] could be confused by small dimensions on fragmentary material because, although early Palauans are lineally normal in height, the post-cranial skeletal elements are gracile and retain small dimensions throughout. Using skeleton -001 as an example, we are presented with a female in her early 20s (based on dental development and wear, epiphyseal closure, and auricular morphology). Although ‘gracile’ throughout her entire skeleton, muscle development (as exhibited by linea aspera and gluteal line development as well as deltoid tuberosity rugosity and definition), would be classed as moderate and about the expected condition for a female of this developmental age living in this environment.

One point we feel needs addressing at this juncture revolves around the question of what constitutes small body size in modern humans. The idea that female height of between 152 and 157 cm constitutes short stature is false. Migliano et al. [62] report an average female height of 159.9 cm (from a database of 434 ethnographic populations) and an average female pygmy size of 140 cm. In addition, Jungers (personal communication) reports that only one of 38 Andaman Island individuals housed at the Natural History Museum in London has femora longer than the 412 mm recorded for individual -001 from Orrak. Since this is the largest individual in the Andaman sample it is presumably male. In general, prehistoric peoples who practiced a hunter-gatherer/horticultural lifeway tended to be ‘short’ by modern western standards, but are still well within the range of normal sized modern humans. By way of a brief example, average female maximum femoral lengths from the Ancestral Puebloan sites of Arroyo Hondo [63] and Mesa Verde [64] are 400.9 mm (n = 9) and 398.1 mm (n = 10), respectively. Under these circumstances, a female with a height of between 152 and 157 cm would be considered average for modern humans practicing a traditional lifeway, not small.

### Cranial size and Development

**Figure 4:**
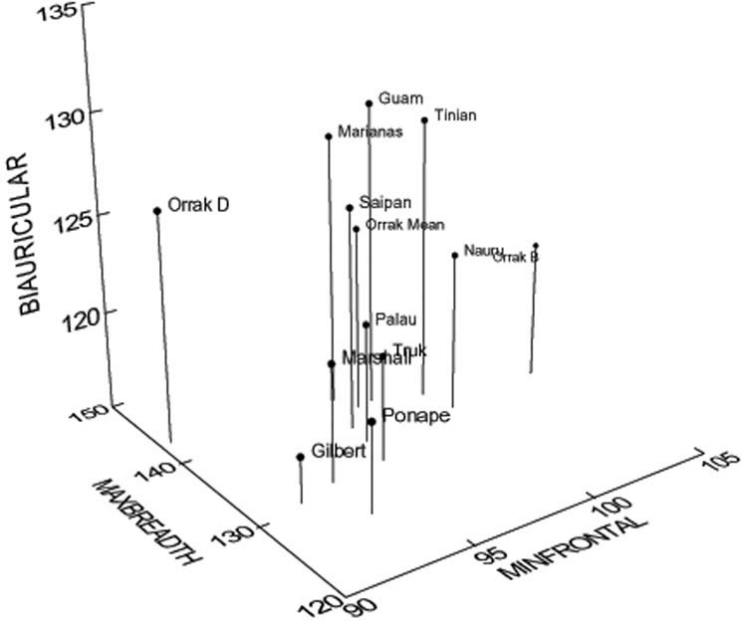
3D scatterplot of Orrak cranial measurements compared to other Micronesian male samples. Note small minimum frontal breadth for Orrak D [Comparative data from 10], [23].

**Figure 5:**
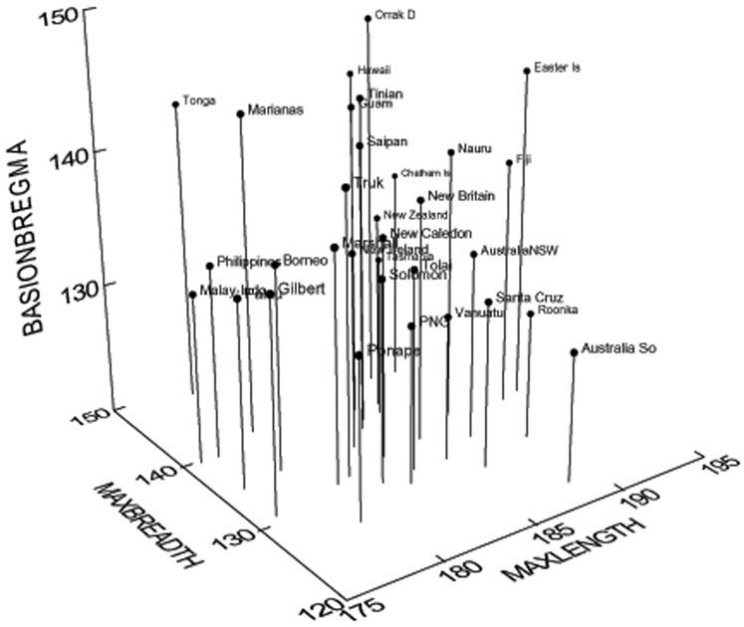
3D scatterplot of Orrak cranium D compared to western Pacific and oceanic male samples. Maximum cranial breadth, maximum length and basion-bregma height produce a good overall view of cranial size and show that Orrak D is large [Comparative data from 10], [23], [98].

Berger et al. [1] claim that reduced cranial dimensions, primarily based on isolated frontal bones, indicate reduced cranial size, possibly as small as that of *H. floresiensis* and well below that expected for normally sized modern humans. Taken in isolation, a small frontal (e.g., small minimum frontal breadth) may seem a good proxy for determining cranial size. However, when considered in light of dimensions found in other early Palauans not considered by Berger et al. [1], small frontal dimensions are not as aberrant as they first appear. Relatively small minimum frontal breadths, on the order of 90 mm [3] to 96 mm [10] (based on 14 adult male crania) appear to be common among early Palauans and are part of a cranial morphology that includes relatively large maximum lengths and breadths and normal cranial capacity. For example, specimen Orrak D (see Figures 4 and 5) presents measurements of: Minimum frontal breadth, 90.5 mm; Maximum cranial length, 187 mm; Maximum cranial breadth, 143 mm; basion-bregma height, 148 mm. Basing cranial capacity on three, unnamed, facial dimensions taken from fragmentary remains does not even warrant comment. However, if the faces of early Palauans were, indeed, relatively small, but attached to normal sized neurocrania, measurements of the face alone would lead to an underestimation of true cranial capacity.

One final comment must be made concerning Berger at al.'s [1] attempts to determine cranial capacity. On page 8 they note that “[t]he only crania complete enough to allow determination of endocranial volume are heavily encased in flowstone, which has deterred our best efforts to estimate brain size in the Palauan sample (see Supplementary data S5).” The flowstone covered cranium depicted in this photograph appears to be that of a child of approximately 4–5 years of age. Based on their shape and placement within the dental arcade, the two right molars remaining in the maxilla are clearly deciduous and the entire morphology of the face and cranial vault is that of a child. It should be noted that *all* other cave/rockshelter burial sites found in the Rock Islands of Palau have a wide demographic profile that includes numerous subadults. Interestingly, Osborne [13, p. 436] specifically mentions Omedokel (Eil ra Rechiklau) as being a location where a *Rubak* (elder Palauan male) was “said to have buried a child there.” Osborne [13, p. 436] goes on to say: ”[i]t appeared from the bones noted and examined that only the bodies of younger persons were placed in the cave…The burial that is generally remembered in the cave is, of course, that of Rubak Rechiklau's child.”

So, it comes as no surprise that skeletal remains from children would be found at this or other burial sites in Palau. Indeed, Berger et al. [1, p. 4] note that they have subadults present in their fragmented assemblage, but that “all specimens analysed in this paper exhibit skeletal or dental indicators of adult developmental age (see Supplementary Data S2, S3).” Yet, nowhere in the paper or the supplementary data does it indicate what methods they used for determining age-at-death of any of the material they examined. Berger et al. [1] simply assume that this skull must be of a small-bodied individual because of their calculations which, as we have demonstrated, are misinterpreted for a number of reasons. Given that many of the remains, including this skull, are encrusted with carbonate precipitate, determining age-at-death may be impossible.

In addition, when cranial measurements of the Orrak samples are compared to other Micronesian (Figure 4) and western Pacific and Oceanic samples (Figure 5), it is readily apparent that they fall within the range of other known populations, with one specimen (Orrak D) actually falling on the larger end of the spectrum. Finally, preliminary measures (frontal and occipital chords and maximum cranial breadth) of three crania recovered during the Fall 2007 excavations that are currently undergoing preparation are well within the range of modern human cranial size [61].

### Dental Metrics and Megadontia

Among the claims of Berger et al. [1] is that the dental dimensions of these early Palauans classify as megadont which they note is common in small bodied hominins such as *Australopithecus* and *H. floresiensis*. They report bucco-lingual dimensions that, although large, fall within the range of dental dimensions reported for early Pacific Island samples [20], [22], [61], [65]. As with their observations concerning body size, Berger et al. [1] appear to be unaware of the large body of comparative dental metric data available for East Asia, Southeast Asia, Indonesia, the Bismarck Archipelago, and Oceania in general [e.g., 20], [22], [61], [65]. Had they compared their scant dental metric data with those of other regions in the Pacific, or elsewhere in the world, they would have seen that large teeth are not uncommon in early (pre-2000 BP) peoples of these regions. Berger et al. [1] do include a modern human sample in their limited comparative data set; this is a “global *H. sapiens* male and female sample” taken from Brown et al.'s [66] supplementary material, but is completely unprovenienced and has no sources listed. Teeth are only megadont if the people are small—if the people are normal sized, then the teeth are just big (and that is not unusual).

### Third Molar Agenesis

Berger et al. [1] state that third molar agenesis is the norm and that when erupted they are “always malrotated” (p. 8). We have three complete dentitions from Orrak; one with all third molars erupted and occluding normally, one with all third molars agenic, and one with a normally erupted lower right third molar, an impacted lower left third molar, and agenesis of the upper third molars. In addition, one isolated maxilla is agenic for third molars and one is not. From this, no trends can be discerned and no conclusions drawn concerning the frequency of third molar agenesis among early Palauans. However, since the third molar is the most frequently agenic tooth and agenesis of one or more molars can reach frequencies greater than 30% in some populations [67], the possibility that third molar agenesis is common in an island population would not be a surprise. As Douglas et al. [68, p. 297] noted from a relatively large skeletal series from the prehistoric Chamorro site of Apurguan on Guam, agenesis of the third molar is common in both sexes. Island populations tend, by their nature, to be more endogamous, so dental traits that appear to be hereditary, such as third molar agenesis, could easily exhibit relatively high frequencies [69].

### Mandibular Morphology

Currently we have three complete mandibles from Orrak. Although they are still undergoing preparation at this time, all appear to exhibit average dimensions. In addition, although not terribly robust, all possess well developed mental eminences and do not evince the dental crowding Berger et al. [1] claim is common. Malocclusion, known anthropologically as occlusal variation, exhibits a range of manifestations, the most common of which is crowding of the anterior mandibular dentition [70]. Extreme anterior crowding is not unusual on an individual basis even in panmictic populations, so to even imply that it is some kind of populational marker reveals a lack of understanding of the etiology and variability of occlusal variation across populations.

### Summary of Skeletal Material

Overall, a review of the literature on prehistoric Palauan burials and our analysis of the skeletal assemblage at Chelechol ra Orrak indicate that the postcranial, craniofacial, dental, and mandibular measurements are all from normal sized individuals for this region. In addition, the traits that Berger et al. [1] claim reflect a primitive condition are not found to be so when considered in light of a more comprehensive understanding of the morphological variation of more complete and well documented skeletal series from Palau. The point is that extreme reduction in body size, regardless of the mechanism, does not need to be employed to result in the suite of morphological traits recorded for some early inhabitants of Palau. Our data indicate that, particularly for females, small articular dimensions are not indicative of small body size, but instead follow a pattern of generally gracile skeletal morphology.

### Insular Dwarfism in Palau?

Although the above comparisons clearly refute the claims by Berger et al. [1] on a biological basis, what about their argument that the Palauan archipelago is an environment conducive to rapid insular dwarfing in *Homo sapiens*? Berger et al. [1, p. 1] state that “dwarfing” is in response to “the combined factors of relative genetic isolation, a reduced resource base, hot and humid climates, hilly topography, thick undergrowth of vegetation, and (in certain island contexts) an absence of terrestrial predators.” This follows traditional hypotheses of why pygmy populations may have evolved such as locomotion in dense forests, thermoregulation, exposure to tropical diseases, poor nutrition, or endurance against starvation [see 62], but as Migliano et al. [62, p. 20218] argue, “the small body size of African and Asian pygmy populations evolved independently as a case of evolutionary convergence, resulting from a life history tradeoff between the fertility benefits of larger body size and the costs of late growth cessation under the circumstance of significant young and adult mortality.”

Berger et al. [1, p. 1,2] go on to say that “the islands of Palau are devoid of indigenous terrestrial mammals and large reptiles, and prehistoric subsistence economies were based on swidden agriculture and the utilization of marine resources” and that “[f]irm archaeological evidence of fishing primarily from near shore and lagoonal habitats, dates to only about 1700 years ago, although further sampling of early sites is likely to push this date back in time.” Because the authors infer that these conditions led to smaller-bodied individuals in Palau and that they “exemplify the regularity with which small body size—physiological dwarfing—emerges in island contexts”, suggesting too that the “traits observed in the Palauan sample are seen also in specimens from Flores” (9), it is necessary to address each of these issues separately.

### Resource Availability

Early Palauans were confronted with a virtual cornucopia of marine resources that was supplemented with taro and other plant and animal foods brought in by colonists or exploited locally [e.g., 8], [15], [50], [71]. A number of faunal assemblages from archaeological sites clearly demonstrate this, as does recent stable isotope analysis of early (ca. 2300–3000 BP) human bones from Chelechol ra Orrak and Ngermereues Ridge that show a high percentage of marine foods in the diet (with additional intake of terrestrial plants) [72]. To this might also be added isotope results from residues from a 2200 year old pot excavated from Ulong Island that demonstrate cooking of marine and terrestrial foodstuffs [18].

A related oversight by Berger et al. [1] is that the contribution of marine foods in the diet necessitates calibrating radiocarbon dates of human bone to reflect this consumption. Berger et al.'s [1: Supplementary Data 1] reported dates presume a wholly terrestrial diet. If, for example, these individuals' diet consisted of 40% marine foods, the dates could actually be 250 years younger (i.e., 2770–2500 cal. BP for sample B:OR-14:8-1200 instead of 2890–2750 cal. BP at 2σ), suggesting that there is an even wider chronological gap between these and the Orrak samples.

It is noteworthy that Berger et al. [1] cite Fitzpatrick and Kataoka [8] as the earliest known evidence of fishing in Palau (dating back to ca. 1700 BP)—this is incorrect. As quoted on the first page of this paper, “[r]ecent excavations by Fitzpatrick [7] in the northern Rock Islands reveal that substantial fish remains date back to at least 1700–1600 BP with fishbone present in even lower strata dating to ca. 3000 BP” [8, p. 1]. Additionally, Clark's (2005) work on Ulong established that inshore reef species of fish and large shellfish, particularly *Tridacna* and *Hippopus*, were important foods by ca. 3000 BP [see also 47], but this information is not cited by Berger et al. [1].

Regardless of this oversight, let us say, for example, that Berger et al.'s [1, p. 10] comment that the features supposedly observed on skeletal specimens from Ucheliungs and Omedokel “may best be explained as correlates of small body size in an island adaptation, regardless of taxonomic affinity”, in part or in whole because of an impoverished resource base. Berger has also stated this in various media outlets, noting in one that “[t]here were at the time no large terrestrial animals so it is likely that the early Palauens [sic] had to survive on only near shore marine resources. While this island looks like a Paradise these early people, who may have been stranded, were really living under a great deal of dietary stress” [73].

We find it difficult to fathom why this would occur in such a biologically rich and diverse archipelago as Palau, particularly over such a short span of time (hundreds of years) and with archaeological evidence throughout the Rock Islands clearly suggesting otherwise [8], [15], [42], [74]. Berger et al. [1] are either ignorant of, or have selectively excluded, the vast amount of literature testifying to the diversity and abundance of marine, freshwater, and terrestrial ecologies in Palau and the Indo-Pacific in general that were exploited prehistorically [e.g., see 33]–[35], [38], [39], [75], [76] and/or assume wrongly that: 1) larger vertebrates are required to maintain a viable population; and 2) that marine resources are insufficient to be a major food source. Decades of archaeological research worldwide, including several important sites in South Africa (which has the earliest evidence for modern humans exploiting marine resources dating back to 160 kya) [see e.g., 77], [78], has shown this to be very misguided assumption [see 79], [80 for detailed discussions of early human use of aquatic resources].

Omedokel and Ucheliungs caves, as the authors acknowledge, do not appear to have evidence of long-term, intensive occupation, strongly suggesting that the remains were brought to the caves after death. As Fitzpatrick and Nelson [2] and others (including tourists) have noted throughout the Rock Islands of Palau, this is commonplace and also the earliest form of mortuary behavior. As such, we would posit that stable isotope analysis of human bone from these two sites would reflect a mixed diet of marine and terrestrial foods, similar to what other similarly aged burials have revealed. It is reasonable to assume then that peoples buried in these caves also had access to terrestrial foods that could only be grown on the larger, mostly volcanic islands such as Koror and Babeldaob, came into contact with other Palauan villages, or more likely, that they themselves were members of these same groups.

It is also important to note that the Palauan islands, although scattered across 30 km of ocean, are nearly all intervisible from one to another and found within a barrier reef system that creates a buffer from the open ocean, allowing for canoes to travel between them even in fairly rough conditions. We think it highly unlikely that people who buried their dead at Ucheliungs and Omedokel, even if they were not living on one of the larger volcanic islands (which afforded greater access to terrestrial foods and agricultural land), would not have seen or interacted with other people over even a short period of time. This was clearly the case on Ulong where the ceramic temper of the oldest pottery and associated lithics (andesite and ironstone) derive from the main island [15]. As we have also noted on our numerous trips to Rock Islands and the northern atolls, pottery is frequently found at sites in caves and along beaches—this required clay to produce, which is only found in sufficient quality and quantity on the larger volcanic islands (i.e., people were frequently traveling between all of the hundreds of islands through time). The obvious question arises as to why, if peoples became dwarf-like over time because of a reduced resource base, relative genetic isolation, etc., did it not occur in many other parts of the Pacific where islands such as Rapa Nui, Tasmania, the “mystery” islands and numerous coral atolls which were, in comparison to Palau, much more isolated and resource impoverished? The islands in East Polynesia, for instance, are among the most remote and, in environmental terms, resource-impoverished landmasses in the world, yet despite these conditions there is no evidence on any Polynesian island for a trend to small-body size in prehistory [e.g., 81], [82].

Berger et al. [1] also state that “archaeological evidence indicates that inhabitants of Palau were in contact with their neighbors on other Western Carolinian islands but by the time of European contact, Palauans were no longer engaged in voyaging to distant islands.” This may or may not be true—but the fact remains that other islanders do appear to have ventured to Palau from Yap, perhaps aided by sailors from the coral outer islands, to quarry stone money before direct European contact [83], [84]. The antiquity of such activities is still under investigation, but the point is that it does not take Palauans leaving Palau for contact with other peoples to occur.

### Continuity and Discontinuity in the Archaeological Record

From ca. 2890–940 cal. BP (or even later if calibrated correctly), Berger et al. [1] suggest that a group of small-bodied people were living in Palau and that the body size of Palauans increased after 940 cal BP. However, research in Palau to date indicates that people of normal size occupied the archipelago between 2000 and 3000 years ago which contrast with the findings of Berger et al. [1]. Our research presents us with three scenarios for the population structure in prehistoric Palau prior to approximately 1000 BP. First, a small-bodied population of humans occupied Palau to 940 BP when they were replaced by a population of normal sized people, either through immigration (scenario 1a) or *in situ* evolution (scenario 1b). Second, a single, normal sized, population inhabited the archipelago from at least 3000 BP. And third, two populations that did not interbreed—one small-bodied and the other normal sized—occupied the islands until 940 BP when the small-bodied group died out or was absorbed by the other.

The fact that each of the above scenarios would leave distinct traces across the landscape raises the question: what does the archaeological record show to support each of these scenarios and/or what would one expect the archaeological record to show under each scenario? For scenario 1a, where an immigrant population of normal sized individuals replaces a small-bodied people around 1000 BP, the archaeological signature would likely be a discontinuity showing the replacement of one culture with another. This could include new pottery composition and style, different housing types, differential land and resource base use, and a dissimilar tool kit. With scenario 1b, in which the resident small-bodied population evolves into normal sized descendents, the archaeological record would show some of these same discontinuities, but would most likely center on differential land and resource use. This is implied because Berger et al. [1] contend that a depauperate resource base or underutilization of available resources led to insular dwarfing. If the resident population of small-bodied people underwent a rapid shift in stature and body mass, there must also have been a shift in resource base utilization and this would be visible in the archaeological record.

A single population of normal sized people inhabiting Palau over an extended time, as in scenario 2, would most likely follow a standard pattern of gradual change in cultural markers and land and resource use. This could manifest as changes in housing style, habitation patterns, burial practices, and agricultural intensification as population increases. This scenario would produce general continuity in the archaeological record with any changes reflecting ancestor-descendent relationships. On the other hand, scenario 3, where two separate populations occupy Palau from 3000–940 BP, would leave an archaeological signature characterized by discontinuity. If each people inhabited different areas of the archipelago, one would expect the archaeological record to reflect this by showing that two cultures existed separately as they would leave different markers on the landscape, particularly in the realm of land and resource use. Because of the limited space within the Palauan archipelago, one could also expect there to be a shift in the archaeological record as populations occupy and reoccupy areas over time. This would lead to site based discontinuity reflecting alternating use of sites and associated resources.

In this section we briefly review data for continuity/discontinuity in the archaeological record of Palau to see which scenario it most closely resembles. Because of its importance to any hypothesis involving a small-bodied population, we examine the archaeological and historical evidence for continuity/discontinuity in Palau's past, particularly the period around 1000 BP to see which of our three scenarios it most closely resembles.

### Settlement patterns

Between 1600 BP and 800 BP, the landscape of Palau was gradually modified by upland earthwork constructions, culminating in the monumental terraces and creation of ‘crown and brim’ hilltops, after which people began to build villages with stone architecture [19], [85]. Phear [86, p. 138], in a detailed examination of this settlement-pattern shift in Ngaraard (northern Babeldaob), views the creation of formalized village space after 1000 BP as the result of internally generated change from population increase. This was coupled with the need for villages to control lowland resources, both of which suggest an increase in inter-group conflict for which there is traditional and archaeological evidence [87], [88]. The burial context also follows the move seen in the formalized village pattern, with interments placed in earthworks prior to 1000 BP, followed by burial in stone platforms representing clan/lineage house foundations after ca. 1000 BP [2]. Several villages in the Rock Islands were abandoned 500–600 years ago, possibly as a result of climate change [89], while Palauan traditions mention community relocation as the result of warfare [17].

### Material culture

Pottery was made in Palau continuously from 3000 years ago, and the ceramic sequence shows several changes [e.g., 15], [50], the most marked of which took place 2500 years ago and involved the use of fired clay (grog) temper in the manufacture of a thin-walled jar with a short everted rim. Around 950 years ago there is another significant change with the use of thick-walled bowls with inverted flange rims, although the change in vessel form is accompanied by continuity in the use of grog temper and the use of clays with a high organic content [15]. The thick-walled flange rim vessels have been likened to Type X ceramics dating to 1000 BP from the Huon Peninsula-Siassi Islands of Papua New Guinea by Specht et al. [90]. Some of the Type X pottery has a grog temper, but several vessel forms and decorative techniques and designs [90: Fig. 4] are unlike anything yet found in Palau [9], [91]. Rather than a movement from Papua New Guinea to Palau, Specht et al. [90, p. 38] see a movement from Palau to Papua New Guinea to explain the ceramic similarities between Type X and late-prehistoric Palauan ceramics. No exotic artifacts from beyond Palau, such as non-local ceramics and stone tools that might support the idea of a major migration to Palau around 1000 BP, have been found in any archaeological investigations to date.

### Subsistence strategy

The Austronesian expansion to the islands of Remote Oceania in prehistory is associated with a mixed economy involving transported economic plants and animals combined with the harvesting of wild foods, particularly marine resources. Palau was no different, except that some domestic animals such as the pig and dog were either not introduced or did not survive. Two important economic plants, coconut (*Cocos nucifera*) and betel nut (*Areca catechu*), are probably indigenous to Palau [92] (with Palauans chewing betel nut by at least 3000 years ago; [see 50]], while direct evidence for the introduced swamp taro (*Cyrtosperma*) has been dated to 3000 BP or earlier [56, p. 171]. Indirect indicators of agriculture include extensive land clearance, earthwork terracing, and abundant ceramics, some with carbonised food remains, in both inland and coastal locations [18], [19], [49], [55]. Intensification of taro (*Colocasia esculeta*) production from the creation of lowland pond-field systems is associated with the development of stone-work villages and the increasing control and competition for resources. Along with introduced and indigenous horticultural crops, Palau's extensive marine resources of fish and shellfish were utilized for over 3000 years [8], [15], [47], [50].

### Linguistic studies

Palauan, along with Chamorro, are the only languages in Remote Oceania that are not classified as ‘Oceanic’, and the two are instead grouped with Western Malayo-Polynesian languages found from the Malay Peninsula through Indonesia, Sulawesi, and the Philippines [93]. The Palauan language is considered to be an isolate remaining from an early movement out into the Pacific ca. 4500–3000 years ago prior to the formation and spread of Oceanic, and while some recent borrowing from Yap is apparent [see also 49, p. 127], there is no data to suggest that the original language has been affected by the arrival of a migrant culture [94], [95].

### Summary

Berger et al. [1] propose that Palauan stature increased from small-bodied ‘pygmy’ to larger-sized people at ca. 1000 BP, but they did not specify the cause for such a dramatic change. Archaeological and linguistic data were reviewed to see whether external or internal factors that might account for the degree of stature change could be identified. The evidence for migration was scrutinized because environmental and subsistence shifts do not appear sufficient to account for the magnitude or rapidity of the proposed change in stature. In short, the archaeological sequence and linguistic history of Palau, suggests cultural and population continuity, with Palauan society modified over time from ongoing developments in economic and socio-political spheres, along with the effects of local and long-distance interaction.

## Discussion

In a sense, we have used a “sledgehammer to crack a nut” by detailing numerous lines of evidence to refute narrowly constructed research that obviously had extensive methodological and analytical flaws. While some may see the Berger et al. [1] paper as being so egregious that few will take it seriously (and as such, does not necessitate the lengthy response we have presented here), we feel that it is extremely important for the scientific community and laymen alike to be fully aware that the data described by Berger et al. [1] is fundamentally flawed and does not mesh with the known biological and archaeological data from Palau. The wide media exposure given to the Berger et al. [1] paper is also of major concern—a lack of response by scholars more familiar with Oceanic prehistory and modern human variation might be seen as support for the hypothesis that small-bodied humans are indeed found in Palau.

As we have illustrated, newly and previously collected data from Chelechol ra Orrak indicate that early Palauans were of normal size and that morphological characteristics such as small articular and facial dimensions and large teeth found in these individuals are well within the variation seen in modern human populations—they are not primitive traits that reflect “pliotropic [sic] or epigenetic correlates of developmental programs for small body size” [1, p. 3]. We find no evidence skeletally or archaeologically to support the claims by Berger et al. [1] that there was a “small-bodied” population in Palau resulting from insular dwarfism or even that there was a new, separate, and isolated, migratory group.

The small and scattered skeletal assemblage from Ucheliungs and Omedokel caves may show traits that would appear to be “primitive” to the genus *Homo*, but comparisons with other Palauan samples and those from both within and outside of the Pacific show them to fall well within the range of modern human variation. As most paleoanthropologists and osteologists who study variation within anatomically modern *Homo sapiens* know, individuals of any ancestry can have large supraorbital tori, an absence of chins, smaller brain sizes, and generally smaller stature.

We also find no support that “[t]he modern human skeletal remains from Palau, in conjunction with pygmoid populations across Australasia, exemplify the regularity with which small body size–physiological dwarfing–emerges in island contexts…” [1, p. 9], at least in the case of humans. Research on insular dwarfism in numerous species, including mammals indicates, however, that “[w]hile the most extreme examples are highly compelling, they do not show the enormous variation characterizing the pathways of insular size evolution and do not amount to a general rule” [96]. Although *Homo floresiensis* may indeed be a case of a species succumbing to the effects of dwarfism in an island context, we would like to point out that Flores was colonized around 800–900 thousand years ago by early *Homo* [97], [98] who had not developed sophisticated watercraft and navigational techniques as is seen in the Pacific 3000–4000 years ago and that the dates for *H. floresiensis* span a long temporal range from 90–18,000 years ago. This would support the notion that if insular dwarfism were to occur in *Homo*, it would have required an island population to have been fairly or completely isolated for tens of thousands of years—this is certainly not the case for Palau.

Archaeological and linguistic data demonstrate that there was a high level of continuity in Palau prehistorically, with no evidence to suggest that a new migratory group arrived, lived in isolation, and then was later absorbed into a normal sized population prior to European contact. There are also no discernible shifts in subsistence or environment that would account for a dwarf-like population to have evolved *in situ*. In sum, current physical anthropological evidence does not support the claims by Berger et al. [1] that human remains found at Ucheliungs and Omedokel caves are from a small-bodied population that fall outside the realm of normal modern human variation in Palau or elsewhere. Nor are their findings supported based on what is found in the archaeological, paleoenvironmental, linguistic, or historical records. As such, the results of Berger et al. [1] should be carefully scrutinized in the face of comparative data that strongly suggests otherwise and which requires independent verification by other scholars who are more familiar with Pacific Island prehistory and modern human variation.<</p>

## References

[1] Berger LR, Churchill SE, De Klerk B, Quinn RL (2008). PLoS ONE.

[2] Fitzpatrick SM, Nelson GC (2008). From limestone caves to concrete graves: 3000 years of mortuary practice in the Palauan archipelago of western Micronesia.. Intl J Osteoarchaeol.

[3] Nelson GC, Fitzpatrick SM (2006). Preliminary investigations of the Chelechol ra Orrak cemetery, Republic of Palau: I, skeletal biology and paleopathology.. Anthropol Sci.

[4] Rieth TM, Liston J (2001). Archaeological Data Recovery at Ngermereus Ridge, Ngesaol, Koror, Republic of Palau..

[5] Fitzpatrick SM (2002). AMS dating of human bone from Palau: New evidence for a pre-2000 b.p. settlement.. Radiocarbon.

[6] Fitzpatrick SM, Boyle J (2002). The antiquity of pearl shell (*Pinctada* sp.) burial artifacts in Palau, Western Micronesia.. Radiocarbon.

[7] Fitzpatrick SM (2003a). Early human burials in the western Pacific: Evidence for a c.3000 year old Occupation on Palau.. Antiquity.

[8] Fitzpatrick SM, Kataoka O (2005). Prehistoric fishing in Palau, Micronesia: evidence from the northern Rock Islands.. Archaeol in Oceania.

[9] Osborne D (1979). Archaeological test excavations Palau Islands 1968–1969.. Micronesica Supplement.

[10] Pietrusewsky M (1990a). Craniometric variation in Micronesia and the Pacific: a multivariate study.. Micronesica Supplement.

[11] Pietrusewsky M (1990b). The physical anthropology of Micronesia: a brief overview.. Micronesica Supplement.

[12] Pietrusewsky M (1990c). Craniofacial variation in Australasian and Pacific populations.. Am J Phys Anthropol.

[13] Osborne D (1966). The Archaeology of the Palau Islands. Bernice P. Bishop Museum Bulletin 230.

[14] Blaiyok V (1993). Archaeological Survey of Airai State, Republic of Palau..

[15] Clark G (2005). A 3000-year culture sequence from Palau, Western Micronesia.. Asian Perspectives.

[16] Clark G, Wright D, Sand C (2003). The colonisation of Palau: Preliminary results from Angaur and Ulong.. Pacific archaeology: assessments and prospects. Les Cahiers de l' Arche'ologie en Nouvelle-Cale'donie 15. New Caledonia.

[17] Clark G, Wright D (2005). On the periphery? Archaeological investigations at Ngelong, Angaur Island, Palau.. Micronesica.

[18] Clark G, Wright D, Anderson A, Green K, Leach F (2007). Reading Pacific pots.. Vastly ingenious: the archaeology of Pacific material culture in honour of Janet M. Davidson.

[19] Liston J, Mangieri TM, Grant D, Kaschko MW, Tuggle HD (1998). Archaeological data recovery for the Compact Road, Babeldaob Island, Republic of Palau. Historic Preservation Investigations, Phase II. Volume II: Fieldwork Reports..

[20] Brace CL, Hinton R (1981). Oceanic tooth-size variation as a reflection of biological and cultural mixing.. Current Anth.

[21] Kirch P, Swindler D, Turner C II (1989). Human skeletal and dental remains from Lapita sites (1600–500 BC) in the Mussau Islands, Melanesia.. Am J Phys Anth.

[22] Brace CL, Brace M, Dodo Y, Hunt K, Leonard W, Yongyi L, Sangvichien S, Xiang-Qing S, Zhenbiao Z (1990). Micronesians, Asians, Thais, and relations: a craniofacial and odontometric perspective.. Micronesica Supplement.

[23] Hanihara T (1992). Dental and cranial affinities among populations of East Asia and the Pacific: The basic populations in East Asia, IV.. Am J Phys Anth.

[24] Pietrusewsky M, Sargat L, Blench R, Sanchez-Mazas A (2005). The Physical Anthropology of the Pacific, East Asia, and Southeast Asia: a multivariate craniometric analysis.. The peopling of East Asia: putting together archaeology, linguistics, and genetics.

[25] Pietrusewsky M, Simanjuntak T, Pojoh IHE, Hisyam M (2006a). The initial settlement of remote Oceania: the evidence from physical anthropology.. Austronesian diaspora and the ethnogenesis of people in the Indonesian Archipelago.

[26] Pietrusewsky M, Oxenham MR, Tayles N (2006b). Chapter 3. A multivariate craniometric study of the prehistoric and modern inhabitants of Southeast Asia, East Asia, and surrounding regions: a human kaleidoscope?. Bioarchaeology of Southeast Asia.

[27] Pietrusewsky M, Katzenberg MA, Saunders SR (2008). Metric analysis of skeletal remains: methods and applications.. Biological anthropology of the human skeleton, (2^nd^ editorion).

[28] Pietrusewsky M, Douglas MT, Ikehara-Quebral RM (1997). An assessment of health and disease in the prehistoric inhabitants of the Mariana Islands.. Am J Phys Anthropol.

[29] Sem G, Underhill Y (1994). Implications of Climate Change and Sea Level Rise for the Republic of Palau. Report of a Preparatory Mission, South Pacific Regional Environmental Programme.

[30] Corwin CG, Rogers CL, Elmquist PO (1956). Military geology of Palau Islands.

[31] Johannes R (1981). Words of the Lagoon: Fishing and Marine Lore in the Palau District of Micronesia.

[32] Maragos JE, Kepler AK, Hunter-Anderson RL, Donaldson TJ, Geermans SJ, McDermid KJ, Idechong N, Patris S, Cook C, Smith B, Smith R, Meier KZ (1994a). Synthesis report: Rapid ecological assessment of the Southwest Palau Islands of Palau..

[33] Maragos JE, Cook CW (1995). The 1991–1992 rapid ecological assessment of Palau's coral reefs.. Coral Reefs.

[34] Donaldson TJ (1996). Fishes of the remote southwest Palau Islands: a zoogeographic perspective.. Pac Sci.

[35] Donaldson TJ (2002). High islands versus low islands: a comparison of fish faunal composition of the Palau Islands.. Environ Biol Fish.

[36] Maragos JE, Meier KZ, Maragos JE, Meier KZ (1993). Reef and corals of the Southwest Islands of Palau.. Rapid Ecological Assessment of Palau, Part 1: The Southwest Islands of Palau. The Nature Conservancy, Honolulu.

[37] Myers RF (1999). Micronesian reef fishes, 3rd ed.

[38] Donaldson TJ, Myers RF (2002). Insular freshwater fish faunas of Micronesia: patterns of species richness and similarity.. Environ Biol Fish.

[39] Palau International Coral Reef Center (2007). Coral Reefs of Palau..

[40] Osborne D (1958). The Palau Islands: stepping stones into the Pacific.. Archaeolo.

[41] Lucking LJ (1984). An archaeological Investigation of Prehistoric Palauan terraces..

[42] Masse WB (1989). The Archaeology and Ecology of Fishing in the Belau Islands, Micronesia, Part 1 and Part 2.

[43] Masse WB (1990). Radiocarbon dating, sea-level change and the peopling of Belau.. Micronesica Supplement.

[44] Snyder D, Butler BM (1997). Micronesian Resources Study. Palau Archaeology. Archaeology and Historic Preservation in Palau..

[45] Fitzpatrick SM, Dickinson WR, Clark G (2003). Ceramic petrography and cultural interaction in Palau, Micronesia.. J Archaeol Sci.

[46] Anderson A, Chappell J, Clark G, Phear S (2005). Comparative radiocarbon dating of lignite, pottery, and charcoal samples from Babeldaob Island, Republic of Palau.. Radiocarbon.

[47] Clark G, Anderson A, Wright D (2006). Human colonization of the Palau Islands, Western Micronesia.. J Isl and Coastal Archaeol.

[48] Phear S, Clark G, Anderson A, Sand C (2003). A radiocarbon chronology for Palau.. Pacific archaeology: assessments and prospects. Les Cahiers de l' Arche'ologie en Nouvelle-Cale'donie 15. New Caledonia.

[49] Liston J (2005). An assessment of radiocarbon dates from Palau, western Micronesia.. Radiocarbon.

[50] Fitzpatrick SM (2003b). Shellfish assemblages from two limestone quarries in Palau.. J Ethnobiol.

[51] Clark G (2004). Radiocarbon dates for the Ulong site in Palau and implications for western Micronesian prehistory.. Archaeol Oceania.

[52] Lum JK, Cann RL (2000). mtDNA lineage analyses: origins and migrations of Micronesians and Polynesians.. Am J Phys Anth.

[53] Callaghan R, Fitzpatrick SM (2007). On the relative isolation of a Micronesian archipelago during the Historic Period: the Palau Case Study.. Itl J Naut Arch.

[54] Callaghan R, Fitzpatrick SM (2008). Examining prehistoric migration patterns in the Palauan archipelago: a computer simulated analysis of drift voyaging.. Asian Perspectives.

[55] Athens JS, Ward JV, Stevenson CM, Lee G, Morin F (2001). Paleoenvironmental evidence for early human settlement in Palau: the Ngerchau Core.. Pacific 2000: Proceedings of the Fifth International Conference on Easter Island and the Pacific.

[56] Athens SJ, Ward JV, Attenbrow V, Fullagar R (2004). Holocene vegetation, savannah origins, and human settlement of Guam.. A Pacific odyssey: archaeology and anthropology in the western Pacific: Papers in honour of Jim Specht Sydney: Records of the Australian Museum Supplement 29.

[57] Athens JS, Ward JV (2005). Palau Compact Road Archaeological Investigations, Babeldaob Island, Republic of Palau; Phase I: Intensive Archaeological Survey; Volume IV: Holocene Paleoenvironment and Landscape Change..

[58] Dickinson WR, Athens JS (2007). Holocene paleoshoreline and paleoenvironmental history of Palau: implications for human settlement.. J Island and Coastal Archaeol.

[59] Bedford S, Spriggs M, Regenvanu R (2006). The Teouma Lapita site and the early human settlement of the Pacific Islands.. Antiquity.

[60] Auerbach BM, Ruff CB (2004). Human body mass estimation: A comparison of “morphometric” and “mechanical” methods.. Am J Phys Anth.

[61] Nelson GC, Fitzpatrick SM (n.d.a). Skeletal remains from early human occupations in Palau (in preparation).

[62] Migliano AB, Vinicius L, Mirazón Lahr M (2007). Life history trade-offs explain the evolution of human pygmies.. Proc Nat Acad Sci.

[63] Palkovich AM (1980). The Arroyo Hondo Skeletal and Mortuary Remains..

[64] Bennett KA (1975). Skeletal Remains from Mesa Verde National Park, Colorado..

[65] Hanihara T (1996). Comparison of craniofacial features of major human groups.. Am J Phys Anthropol.

[66] Hillson S (1996). Dental anthropology.

[67] Brown P, Sutikna T, Morwood MJ, Soejono RP, Jatmiko E, Saptomo W, Due RE (2004). A new small-bodied hominin from the Late Pleistocene of Flores, Indonesia.. Nature.

[68] Douglas MT, Pietruewsky M, Ikehara-Quebral RM (1997). Skeletal biology of Apurguan: a precontact Chamorro site on Guam.. Am J Phys Anthropol.

[69] Nelson GC (1992). Maxillary canine third premolar transposition in a prehistoric population from Santa Cruz Island, California.. Am J Phys Anth.

[70] Nelson GC (1998). Occlusal Variation in Modern India..

[71] Fitzpatrick SM, Donaldson T (2007). Anthropogenic impacts to coral reefs in Palau, western Micronesia during the Late Holocene.. Coral Reefs.

[72] Fitzpatrick SM, Krigbaum J ((n.d.) ). Stable isotope analysis of early human remains from Palau, Micronesia (in preparation).

[73] Patel S (2008). Discovery fuels ‘hobbit’ debate.. http://www.iol.co.za/.

[74] Carucci J (1992). Cultural and natural patterning in prehistoric marine foodshell from Palau, Micronesia..

[75] Brownell RL, Anderson PK, Owen RP, Ralls K (1981). The status of dugongs at Palau, and isolated island group.. The dugong. Proceedings of a seminar/workshop.

[76] Brazaitis P (1992). Recovery plan for the saltwater crocodile, *Crocodylus porosus*, in the United States Trust Territory of the PaciWc Islands, Republic of Palau..

[77] Klein RG, Avery G, Cruz-Uribe K, Halkett D, Parkington JE, Steele T, Volman TP, Yates R (2004). The Ysterfontein 1 Middle Stone Age site, South Africa, and early human exploitation of coastal resources.. Proc Nat Acad Sci.

[78] Marean CW, Bar-Matthews M, Bernatchez J, Fisher E, Goldberg P, Herries AIR, Jacobs Z, Jerardino A, Karkanas P, Minichillo T, Nilssen PJ, Thompson E, Watts I, Williams HM (2007). Early human use of marine resources and pigment in South Africa during the Middle Pleistocene.. Nature.

[79] Yesner D (1980). Maritime hunter-gatherers: ecology and prehistory.. Current Anthropol.

[80] Erlandson J (2001). The archaeology of aquatic adaptations: paradigms for a new millennium.. J of Archaeol Res.

[81] Anderson A (2001). No meat on that beautiful shore: the prehistoric abandonment of subtropical Polynesian islands.. Int J Osteoarchaeol.

[82] Anderson A, White JP (2001). The prehistoric archaeology of Norfolk Island, Southwest 765 Pacific. Records of the Australian Museum, Supplement 27.

[83] Fitzpatrick SM (2003c). Stones of the Butterfly: an Archaeological Investigation of Yapese Stone Money Quarries in Palau..

[84] Fitzpatrick SM (2008). Micronesian Interregional Interaction: Deciphering Multi-Group Contacts and Exchange Systems through Time.. J of Anth Archaeol.

[85] Wickler S, Ladefoged T, Graves M (2002). Terraces and villages: Transformation of the cultural landscape of Palau.. Pacific landscapes: archaeological approaches in Oceania.

[86] Parmentier RJ (1987). The Sacred Remains: Myth, history, and polity in Belau.

[87] Liston J, Tuggle HD, Arkush E, Allen MW (2006). Prehistoric warfare in Palau.. The archaeology of warfare: prehistories of raiding and conquest.

[88] Masse WB, Liston J, Carucci J, Athens JS (2006). Evaluating the effects of climate change on environment, resources depletion, and culture in the Palau Islands between AD 1200 and 1600.. Quaternary Int.

[89] Specht J, Lilley I, Dickinson WR (2006). Type X pottery, Morobe Province, Papua New Guinea: petrography and possible Micronesian relationships.. Asian Perspectives.

[90] Snyder DM (1989). Towards Chronometric Models for Palauan Prehistory: Ceramic Attributes.

[91] Athens JS, Ward JV (1999). Archaeological data recovery for the Compact Road, Babeldaob Island, Republic of Palau..

[92] Pawley A, Zeitoun E, Jenkuei Li P (1999). Chasing rainbows: Implications of the rapid dispersal of Austronesian languages for subgrouping and reconstruction.. Selected papers from the eight international conference on Austronesian linguistics. Academia Sinica 1.

[93] Blust R (2000). Chamorro historical phonology.. Oceanic Linguistics.

[94] Zobel E, Wouk F, Ross M (2002). The position of Chamorro and Palauan in the Austronesian language family tree: Evidence from verb morphosyntax.. The historical and typological development of western Austronesian voice systems. Pacific Linguistics 518, Research School of Pacific and Asian Studies, Australian National University.

[95] Meiri S, Cooper N, Purvis A (2008). The island rule: made to be broken?. Proc R Soc B.

[96] Morwood MJ, O'Sullivan PB, Aziz F, Raza A (1998). Fission-track ages of stone tools and fossils on the East Indonesian Island of Flores.. Nature.

[97] Morwood MJ, Aziz F, O'Sullivan P, Nasruddin, Hobbs DR, Raza A (1999). Archaeological and palaeontological research in central Flores, East Indonesia: results of fieldwork 1997–1998.. Antiquity.

[98] Howells WW (1989). Skull Shapes and the Map: Craniometric Analyses in the Dispersion of Modern Homo..

